# The Section of Oral and Dental Surgery in the International Medical Congress

**Published:** 1885-12

**Authors:** 


### THE SECTION OF ORAL AND DENTAL SURGERY IN THE INTERNATIONAL MEDICAL CONGRESS.

### REPORT OF A SPECIAL CONFERENCE OF DENTISTS HELD AT THE GENESEE HOUSE, IN THE CITY OF BUFFALO, NOV. 16, 1885.

For the purpose of determining the feeling of representative dentists from all sections of the United States, concerning the Dental Section of the Congress of 1887, the following circular, prepared by Prof. Taft, was mailed to about fifty dentists.

SPECIAL CALL.

DEAR SIR—You are no doubt aware of the fact that the difficulties that some time ago interrupted and embarrassed the organization of the Ninth International Medical Congress have been in the main removed, and it is now proposed to complete the organization.

A meeting of the Executive Committee, for this purpose, will be held in New York, on Wednesday, the 18th inst.

That committee consists of the President of the Congress, the Secretary General, the Treasurer, the chairman of the Finance Committee, and the Presidents of the Sections.

The following subjects will be presented for the consideration and action of the committee, viz.:

1. Organization of the Executive Committee.
2. Unfinished business, list of vacancies, etc.
3. Reports of Sub-Committees—Finance, Circulars, Correspondence, etc.
4. Special business. Recognition of the Congress by the United States and foreign governments. Design for a seal, a medal, etc. Public announcement of the organization of the Congress.
5. New business. Appointments of Sub-Committees to report to the Executive Committee at St. Louis. (1) On organizations of Sections and duties of officers of the Congress. (2) On nominations of foreign officers and on vacancies. (3) On foreign papers and discussions.
6. Nominations to fill existing vacancies.
7. Election. Adjournment to day fixed.

The Section of "Oral and Dental Surgery" has been restored to the position assigned it by the original committee.

If the work contemplated in the establishment of this Section is to be done in a manner to obtain the highest results, it is indispensable that it should have the hearty approval and co-operation of the dental profession. In view of this fact, and in order that the opinions of at least some of the more prominent members should be ascertained before the meeting of the Executive Committee in New York, it is proposed to request twenty-five or thirty of the persons most interested in this matter to meet at the Genesee House in Buffalo, N. Y., on Monday the 16th inst., at 10 o'clock A. M., for consultation, suggestions, and a full understanding of the questions involved.

It is earnestly desired and very important that you be present at this meeting, and give such counsel as in your judgment will be for the best interest of our profession.

Yours respectfully,
J. Taft,
W. C. Barrett,
W. W. Allport,
A. M. Dudley.

In compliance with this call, the following named dentists assembled at the time and place designated:

| | |
|---|---|
| J. Taft, Cincinnati, O. | C. N. Pierce, Philadelphia, Pa. |
| A. M. Dudley, Salem, Mass. | W. W. Allport, Chicago, Ill. |
| Geo. L. Field, Detroit, Mich. | J. N. Crouse, Chicago, Ill. |
| C. R. Butler, Cleveland, O. | A. W. Harlan, Chicago, Ill. |
| W. P. Horton, Cleveland, O. | T. W. Brophy, Chicago, Ill. |
| A. O. Hunt, Iowa City, Iowa. | C. R. E. Koch, Chicago, Ill. |
| L. D. Shepard, Boston, Mass. | W. F. Fundenberg, Pittsburgh, Pa. |
| J. B. Coolidge, Boston, Mass. | W. H. Atkinson, New York, N. Y. |
| W. N. Morrison, St. Louis, Mo. | G. C. Daboll, Buffalo, N. Y. |

W. C. Barrett, Buffalo, N. Y.

Letters of regret were received from a number of others, who had been invited.

The meeting was called to order by Dr. Taft, and on motion he was elected chairman, and Dr. Dudley was elected secretary.

Dr. Taft stated the object of the meeting, which was a full consultation and conference as to the action advisable in view of the re-establishment of the Dental Section in the coming International Medical Congress. He gave the present status, and adverted to the coming meeting of the executive committee of the Congress, in New York, Nov. 18th.

*Dr. Crouse*—Said that the first thing to be determined by this conference was the desirability of the establishment of a Section of Dental and Oral Surgery.

*Dr. Shepard*—Enquired why the Section was dropped after once being established ?

*Dr. Taft*—Replied that the only reason that had been given was, that there were too many Sections, and there was not room for them to work.

*Dr. Allport*—Gave a history of the original establishment of the Section, and of his own action in connection with it.

*Dr. Barrett*—Said that those who were in attendance upon the Congress of 1881, in London, had ever since rested under a weight of social and scientific obligation; that they longed for nothing so much as the opportunity to repay it. They hailed with delight the announcement that the Congress of 1887 would be held in this country. When it was announced, they were astounded with the information that no Dental Section was provided for. This, in the country where dentistry had made greatest progress, was to them incomprehensible. If the English physicians had invited us in, was it for America to exclude us ? But soon we were informed that a Section had been established, and a list of officers was submitted. We were content, and began to brace ourselves for the effort of making our Section a yet greater success than that of its predecessor in London. Then came the revolutionary proceedings in the American Medical Association at New Orleans. A single society arrogated to itself the entire direction of the Congress, and proceeded to exclude all who were not in affiliation with it. The old committee was ousted, and our Section was dropped under humiliating circumstances. But the new committee soon found that they had alienated many of the representative men in medicine, and they were met by that which they could but have anticipated. A large number of the officers of the Congress resigned, and declined to have anything further to do with it, and this action was followed by the withdrawal of a considerable portion of the profession from any participation in the organization. A palpable failure stared them in the face, but they were determined to persevere. They saw that the aid of the dentists in this, their extremity, was desirable, and the Section was re-established. But they were now engaged in a heated quarrel, and it seemed demonstrated that the Congress was doomed to a scientific failure. There was no probability that any foreigners of note would attend, and it would lack the character of internationality.

We had been severely snubbed. The secretary had stated that "the omission of the Section of Dental and Oral Surgery was judicious, dentistry not being generally recognized as a legitimate department of medicine." It was only when failure was imminent that they turned to dentistry, and again invited it to come in, and this was done, it had been said, "in response to numerous and urgent requests for its reinstatement, on the part of the dentists." It would be interesting to know who were the dentists who had thus begged to be taken back. Under the present circumstances, unless the original status was resumed, and the original committee reinstated, we could not, unless at the sacrifice of self-respect, again take part. If we were so forgetful of our dignity as to return and attempt what must result in failure, we should remember that we stand, in a certain sense, the representatives of the dentists of the world, and we owe something to them. We have no right to compromise European dentistry by attempting to call such a section "International," when we are fully assured that but a portion of the profession of America, alone, will take part in it. He offered the following resolution:

*Resolved,* That we as members of the dental profession, deem it inexpedient to recommend the organization of a Section of Dental and Oral Surgery in the International Medical Congress of 1887, under the present circumstances.

*Dr. Shepard*—Desired to put apart any consideration of a personal nature. The question is, whether under the present circumstances it is advisable to establish a Dental Section. Will foreign dentists be likely to attend it? He thought not. From New England there is not a single man of acknowledged representative character who will be connected with the Congress. He believes that it will not be of an international character.

*Dr. Allport*—Thought that the differences would soon be healed, and the Congress prove a success.

*Dr. Butler*—Thought it was not to our interests to enter the Congress, under the present circumstances.

*Dr. Koch*—Desired to follow a different line of argument, in opposition to a Dental Section. He thinks that we are taken on sufferance, and for the possible help that we may be able to furnish. We are not invited with that cordiality that warrants our engaging in the Congress. He would prefer to reserve our forces for a possible International Dental Congress.

*Dr. Daboll*—At the meeting of 1881 felt that he but waited the opportunity to contribute something toward the social and scientific entertainment of those to whom he was under a weight of obligation. The Congress of 1887 did not promise such a success as would give him that occasion, and, therefore, he was not in favor of a Dental Section.

*Dr. Hunt*—Thought the resolution did not go far enough. It would be well for us to express the reasons for our action, lest the declaration might be prejudicial to our hopes of such a Section becoming a fixture in future meetings of the Congress.

*Dr. Harlan*—Had a letter from Magitot, in which he stated it as improbable that he should attend, and he thought it extremely unlikely that there would be any foreigners of note present. The dentists formed about one-sixth of those engaged in the healing art, in the United States. Surely something was due them, but it should come in such a way that we could accept it without a loss of dignity. He deemed it inadvisable to enter the Congress of 1887.

*Dr. Brophy*—Had watched the proceedings of the Committee of the Congress with interest, and he believed that unless radical changes were made the meeting could not be a scientific success. It is not possible for our Section to prove a credit to us unless the whole meeting shall be a success, and this was, to say the least, extremely problematical. The dental section of the American Medical Association, that commenced under happy auspices, has dwindled down to almost nothing.

*Dr. Horton*—Does not believe that the best men among the dentists will be willing to be active in the formation of a dental section of the Congress, as it is at present organized.

*Dr. Fundenberg*—Is opposed to the formation of a dental section, for the reasons already stated.

*Dr. Morrison*—Thinks that if we take the course indicated by former speakers, it would indicate that we feared the results. There is a higher position that we might assume. We should keep up the Section for the sake of the future. If we definitely refuse now to take part, as the resolution contemplates, and the Congress should prove a success, we might regret it.

*Dr. Atkinson*—Said that the resolution contemplates no such definite and final refusal. It was only under the present circumstances that we deprecated or declined action. If the organization of the Congress was changed, and those in whom the medical profession placed confidence assumed the direction, we could then act heartily in accord with them.

*Dr. Field*—Fuly concurs with the general tone of the remarks made here. We have been dropped from the Congress under humiliating circumstances, and he is now in favor of staying out. The most dignified course that we can take is to quietly step one side, and watch the progress of the medical fight, ourselves taking no part.

*Dr. Pierce*—Has consulted with prominent medical men of Philadelphia. Dr Hays has received a letter from Sir James Paget, President of the Congress of 1881, and he is in a position to speak for the medical profession of England and Germany, and is positive that no representative men from those countries will attend the Congress of 1887. He believes that we cannot, consistently with self-respect, take any part in the coming Congress.

*Dr. Crouse*—Likes to be on the other side, and to act with the minority, but here he finds himself unable to do so. He does not think that, under the present management, the Congress can prove a success, and believes that we are better out of it.

*Dr. Allport*—Wished to remove any impression that we should be humiliated by entering the Congress. We should here express no opinion, but leave the whole to Dr. Taft, who is a member, *ex officio*, of the Executive Committee, and when he reaches New York he should be allowed to use his own judgment, and if he thinks a Dental Section should be established he should act in conformity with that belief.

*Dr. Shepard*—Said that Dr. Taft had called us together, some of the members of the conference traveling nearly two thousand miles, for the purpose of obtaining our opinion and offering our advice. If no expression of judgment was given the conference was a farce.

*Dr. Coolidge*—Had little knowledge of the subject, and therefore hesitated to express a positive opinion.

The Secretary read letters from Drs. Frank Abbott, J. L. Williams, of Boston, Geo. H. Cushing, Thos. Fillebrown, B. H. Catching, H. J. McKellops, S. B. Brown, Ed. Maynard, W. H. Morgan, W. H. Trueman, C. A. Brackett, E. T. Darby, A. H. Thompson, M. W. Foster and others, the general tone being adverse to the formation of a Section of Dental and Oral Surgery.

*Dr. Dudley*—Said that he was at first anxious for the establishment of such a Section, but when the revolutionary proceedings were adopted at New Orleans, he became convinced that the Congress must result in a scientific failure. If we now took any part in the Congress we shall be connected with the factional fight, and be identified with one of the parties. We cannot with dignity go back when we have once been turned out.

There was considerable desultory discussion regarding the status of such as should be admitted to the Section, if established, and the qualifications necessary for admission.

*Dr. Taft*—Said that a part of the Council of the Section possessed only the D. D. S., and it was not at all probable that any one having that degree, even though he was not a graduate in medicine, would be excluded.

A number of verbal amendments to the resolution were offered, it clearly being the opinion of those present that, under happier auspices, the dentists would heartily co-operate, but all were voted down, as it was urged that the resolution provided for such a contingency.

Finally, the resolution being put to vote, it was adopted unanimously, and, upon motion, the conference adjourned.

